# Editorial: Molecular mechanisms underlying *C9orf72* neurodegeneration, volume II

**DOI:** 10.3389/fncel.2023.1357319

**Published:** 2024-01-08

**Authors:** Jean-Marc Gallo, Agnes Nishimura, Annakaisa Haapasalo

**Affiliations:** ^1^Department of Basic and Clinical Neuroscience, Maurice Wohl Clinical Neuroscience Institute, Institute of Psychiatry, Psychology and Neuroscience, King's College London, London, United Kingdom; ^2^Centre for Neuroscience, Surgery and Trauma, Blizard Institute, Barts and The London School of Medicine and Dentistry, Queen Mary University of London, London, United Kingdom; ^3^A.I. Virtanen Institute for Molecular Sciences, University of Eastern Finland, Kuopio, Finland

**Keywords:** *C9orf72*, autophagy, nucleocytoplasmic transport, telomere, small GTPases, amyotrophic lateral sclerosis, frontotemporal dementia, neurodegeneration

The most common genetic cause of familial and sporadic amyotrophic lateral sclerosis (ALS) and frontotemporal dementia (FTD) is a GGGGCC hexanucleotide repeat expansion in the *C9orf72* gene (Balendra and Isaacs, 2018), defining a group of neurodegenerative diseases collectively referred to as c9ALS/FTD. Three pathological mechanisms have been implicated in c9ALS/FTD: sense and antisense repeat RNA form RNA foci that sequester specific RNA-binding proteins, impairing their normal function (Barker et al., 2017; Swinnen et al., 2020); sense and antisense RNA repeats are translated into the three possible reading frames by repeat-associated non-AUG-initiated (RAN) translation resulting in the production of five dipeptide repeat proteins (DPRs), namely poly-GA, poly-GP, poly-GR, poly-PA and poly-PR. Arginine-rich DPRs (poly-GR and poly-PR) cause severe neurodegeneration in *Drosophila* (Mizielinska et al., 2014) and disrupt the formation of membraneless organelles, including stress granules (Solomon et al., 2021). Finally, haploinsufficiency resulting in reduced levels of the C9ORF72 protein contributes to pathogenesis, albeit not being causative (Xiao et al., 2016).

While DPR toxicity and, to a lesser extent, repeat RNA toxicity, have received a great deal of attention, the goal of our Research Topic is to highlight recent developments on the synergy between the three postulated components of the pathophysiology of c9ALS/FTD, with a special emphasis on the role of *C9orf72* haploinsufficiency, including its implication in the autophagy/lysosomal pathway.

In their review, Diab et al. discuss the normal physiological role of C9ORF72 in the autophagy-lysosome pathway and how it is impaired as the result of *C9orf72* haploinsufficiency. One important aspect covered by Diab et al. review is the interaction between C9ORF72 and Rab proteins, a large group of small GTPases involved in endosomal/lysosomal trafficking. Of note, Alsin, encoded by the *ALS2* gene causing a recessive juvenile form of ALS, is a guanine nucleotide exchange factor for Rab5 (Topp et al., 2004). Lopez-Herdoiza et al. found that decreasing C9ORF72 in mice led to anomalies of the autophagy/lysosomal pathway, substantiating the role of C9ORF72 in autophagy. Knockdown mice displayed cytoplasmic accumulation of the RNA-binding protein, TDP-43, a pathological hallmark of the vast majority of ALS cases and of a high proportion of FTD cases (Neumann et al., 2006), and decreased synaptic density in the cortex. Importantly, these animals also developed behavioral deficits reminiscent of FTD and mild motor phenotypes from 5 months of age. The authors concluded that C9ORF72 partial loss of function contributes to the pathophysiology of c9ALS/FTD.

DPR toxicity and *C9orf72* haploinsufficiency are not mutually exclusive, as shown in the Dane et al. and Bauer et al. studies. To examine the contribution of *C9orf72* haploinsufficiency to DPR toxicity, Dane et al. generated a panel of *C9orf72* homozygous and hemizygous knockout induced pluripotent stem cells (iPSCs) and found that reduced levels of C9ORF72 exacerbated the toxicity of externally applied poly-GR15 in spinal motor neurons differentiated from iPSCs. Treatment with MS023, an inhibitor of Type I protein arginine methyltransferases, partially rescued poly-GR15 toxicity in motor neurons from *C9orf72* expansion carriers or healthy controls.

The endolysosomal pathway plays an important role in c9ALS/FTD (Todd et al., 2023). TMEM106B is a glycosylated, single pass, type 2 transmembrane lysosomal protein; variants of the *TMEM106B* gene have been associated with disease progression in c9ALS/FTD. Bauer et al. showed that knockdown of TMEM106B expression disrupted autophagosome to autolysosome maturation and exacerbated the accumulation of ectopically expressed DPRs in HeLa cells and in astrocytes derived from *C9orf72* expansion carriers. Consistent with the role of the Rab proteins in c9ALS/FTD, as highlighted in the Diab et al. review, lysosomal clustering required Rab7A and coincided with reduced anterograde transport of lysosomes to the cell periphery mediated by the small GTPase, Arl8b. The authors concluded that *TMEM106B* variants may modify c9ALS/FTD by regulating autophagic clearance of DPRs.

As in all ALS cases and most FTD cases, TDP-43 is mislocalized from the nucleus to the cytoplasm and forms abundant cytoplasmic inclusions in affected neurons from *C9orf72* repeat expansion carriers. Disruption of nucleocytoplasmic transport in c9ALS/FTD has been widely demonstrated in a variety of model systems. TDP-43 mislocalization has been linked to nucleocytoplasmic transport defects. In their comprehensive review, McGoldrick and Robertson explore the mechanisms of nucleocytoplasmic transport disruption in c9ALS/FTD. In particular, they highlight the effect of both repeat RNA- and DPR-mediated toxicity on the nuclear pore complex and on components of the nucleocytoplasmic transport machinery, including the Ran-GTPase cycle. Importantly, McGoldrick and Robertson review the evidence that nucleocytoplasmic transport abnormalities in c9ALS/FTD can also be the result of *C9orf72* haploinsufficiency. Finally, the authors conclude their review by discussing the implications of nucleocytoplasmic transport dysfunction in c9ALS/FTD, as well as in non-*C9orf72* forms of ALS/FTD, in the context of TDP-43 mislocalization.

While it is clear that coding variants in a number of genes are disease modifiers or risk factors, these types of genetic variation alone do not explain sporadic ALS, and, considering the strong genetic component of sporadic ALS, other forms of genetic variation are likely to be involved. Al Khleifat et al. examined telomere length in whole genome sequence data from a total of 6,195 samples from Project MinE, an international ALS whole genome sequencing consortium that includes phenotype data. Telomeres maintain DNA integrity during cellular replication; taking into account natural variation with age and sex, Al Khleifat et al. showed a 20% increase in mean telomere length in ALS patients compared to controls (5.5 vs. 5.38 kb), a result validated in brain samples. ALS patients with shorter telomeres had a 10% increase in median survival. Thus, longer telomeres are a risk factor for ALS and worsen prognosis. No difference in telomere length was found between sporadic ALS and familial ALS. Intriguingly, among familial cases, telomere length in *C9orf72* expansion carriers was shorter than in non-*C9orf72* expansion carriers.

Overall, the Research Topic sheds light on the synergy between the three effectors of the pathomechanism of c9ALS/FTD, namely repeat RNA, DPRs and *C9orf72* haploinsufficiency. The pathways highlighted, especially autophagy and nucleocytoplasmic transport, could provide targets for treatment for c9ALS/FTD and possibly for other forms of ALS or FTD.

## Author contributions

J-MG: Writing – original draft. AN: Writing – review & editing. AH: Writing – review & editing.
